# The fronto‐parietal network is not a flexible hub during naturalistic cognition

**DOI:** 10.1002/hbm.25684

**Published:** 2021-10-15

**Authors:** Chiara Caldinelli, Rhodri Cusack

**Affiliations:** ^1^ Trinity College Institute of Neuroscience, Trinity College Dublin Dublin

**Keywords:** dynamic functional connectivity, fronto‐parietal network, movie watching

## Abstract

The fronto‐parietal network (FPN) is crucial for cognitively demanding tasks as it selectively represents task‐relevant information and controls other brain regions. To implement these functions, it has been argued that it is a flexible hub that reconfigures its functional connectivity with other networks. This was supported by a study in which a set of demanding tasks were presented, that varied in their sensory features, comparison rules, and response mappings, and the FPN showed greater reconfiguration of functional connectivity between tasks than any other network. However, this task set was designed to engage the FPN, and therefore it remains an open question whether the FPN is in a flexible hub in general or only for such task sets. Using two freely available datasets (Experiment 1, *N* = 15, Experiment 2, *N* = 644), we examined dynamic functional connectivity during naturalistic cognition, while participants watched a movie. Many differences in the flexibility were found across networks but the FPN was not the most flexible hub in the brain, during either movie for any of two measures, using a regression model or a correlation model and across five timescales. We, therefore, conclude that the FPN does not have the trait of being a flexible hub, although it may adopt this state for particular task sets.

## Graphical abstract

The fronto‐parietal network (FPN) is crucial for cognitively demanding tasks as it selectively represents task‐relevant information and controls other brain regions. It has been argued that it is a flexible hub that reconfigures its functional connectivity with other networks. Using two freely available datasets (Experiment 1, *N* = 15, Experiment 2, *N* = 644), we examined dynamic functional connectivity during naturalistic cognition, while participants watched a movie. Many differences in the flexibility were found across networks but the FPN was not the most flexible hub in the brain. We, therefore, conclude that the FPN does not have the trait of being a flexible hub, although it may adopt this state for particular task sets.

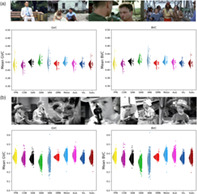

## 1 INTRODUCTION

The fronto‐parietal network (FPN) is crucial for many tasks, and in particular those that are cognitively challenging (Duncan, 2001). One critical role of the FPN is to flexibly represent what is most important about a stimulus or task context (Dehaene, Kerszberg, & Changeux, 1998; Duncan et al., 2000; Freedman, Riesenhuber, Poggio, & Miller, 2001). The FPN also modulates many brain systems (Norman & Shallice, 1986), directing attention by enhancing the most relevant stimulus features or internal representation, which is particularly important when there are distractions. To achieve these, the FPN is highly interconnected with the rest of the brain. These connections have been shown to be important for behavior, with an individual's fingerprint of FPN connectivity predictive of their performance in challenging tasks (Finn et al., 2015).

In recent years, a growing number of studies have examined the dynamic changes in functional connectivity over time (Hutchison, Womelsdorf, Gati, Everling, & Menon, 2013). The FPN in particular shows flexibility in its connectivity, engaging with the brain systems most relevant for the task at hand (Rowe, Friston, Frackowiak, & Passingham, 2002). A recent study by Cole et al. (2013) quantified the adaptability of each brain network, and concluded that a core feature of the FPN is that it forms a “flexible hub” for the brain. They measured functional connectivity of several brain networks during a set of challenging tasks, and found that the connectivity of the FPN to other brain networks was strongly task‐modulated. This shifting pattern of functional connectivity across tasks, supports the idea that regions of the prefrontal cortex adapt to the cognitive task by not only transforming information, but by channeling what is most relevant elsewhere in the brain (Duncan et al., 2000). However, Cole et al. (2013) did not investigate the generality of their conclusion that the FPN is a flexible hub, as brain connectivity was only measured during a set of tasks specifically designed to engage executive functions, with constantly changing complex requirements (Cole et al., 2013). They factorially combined four comparison rules, four sensory features and four response fingers, to give a combination of 64 different tasks (Cole et al., 2013; Ito et al., 2017). For instance, the participants could be presented with two stimuli and be asked to judge if they both had the same color, and to respond with the index finger. As only this task set was examined, it is unclear whether the FPN acts as a flexible hub in general, or whether it only acts as a flexible hub in the context of these challenging tasks.

The current study tests the hypothesis that the FPN exhibits a distinctive trait compared with the other networks, by changing its connectivity in a more flexible way than other networks, in general across many task contexts. We hypothesized that this is an intrinsic characteristic of the FPN, and not a behavior elicited by a particular narrow set of tasks. To test this hypothesis, we characterized the dynamic functional connectivity while participants were engaged in naturalistic cognitive scenarios more representative of everyday life, movie watching. Movies were chosen as they engage us in a similar way to everyday life, in which “the brain makes sense of continuous and complex inputs from the external world” (Spiers & Maguire, 2007), while at the same time satisfying the need to remain motionless in an magnetic resonance imaging (MRI) scanner. We argue that the complexity and concatenation of many scenes into a meaningful plot activate a range of implicit tasks and therefore would require changing patterns of connectivity between brain networks. We applied an analysis pipeline similar to Cole et al. (2013) to two open datasets: the first acquired during the presentation of a full movie (Forrest Gump; Hanke et al., 2014); and the second one during the presentation of a highly engaging short movie by Alfred Hitchcock, which has previously been shown to engage the frontal and parietal areas (Naci, Cusack, Anello, & Owen, 2014).

## 2 EXPERIMENT 1: STUDYFORREST

### 2.1 Methods

#### 2.1.1 MRI data

Experiment 1 used a freely available, high‐resolution functional magnetic resonance imaging (fMRI) dataset from 15 participants recorded at ultra‐high field during prolonged stimulation with an audiovisual film (http://studyforrest.org). The participants underwent an MRI scan while watching a full movie (“Forrest Gump”). Functional MRI data were acquired on a 7‐Tesla Siemens MAGNETOM, and structural images were obtained using a 3‐Tesla scanner. A detailed description of the dataset and data preprocessing can be found in Hanke et al. (2014). All MRI data used in this study were publicly available and anonymized. A proportionate ethical approval was obtained from the ethics committee of the School of Psychology, Trinity College Dublin.

#### 2.1.2 Stimuli

Participants were presented with the movie “Forrest Gump” (R. Zemeckis, Paramount Pictures 1994), with a description for visually impaired people, which was identical to the normal German version of the movie except for narration by a male speaker who describes the visual content of a scene when there is no dialog or other relevant audio content. In order to keep the fMRI sessions under 2 hr, some scenes judged as less relevant to the plot, were removed (for a detailed description of which scenes were deleted see Hanke et al. (2014)). The remaining parts of the movie were then divided into eight segments, each approximately 15 min long. The movie was presented to all participants in full but divided into two different sessions on different days.

#### 2.1.3 MRI acquisition

T1 and T2 structural images were acquired with a 3‐Tesla Philips Achieva equipped with a 32 channel head coil. T1‐weighted images were acquired with the following parameters, 274 sagittal slices (field of view 191.8 × 256 × 256 mm), voxel size was 0.7 mm with a 384 × 384, in‐plane reconstruction matrix; a 3D turbo field echo sequence was used and repetition time was 2,500 ms, inversion time was 900 ms, a flip angle of 8°, echo time was 5.7 ms, and bandwidth was 144.4 Hz/px.

To acquire fMRI, T2*‐weighted echo‐planar images were acquired during audio‐visual stimulation using a whole‐body 7‐Tesla Siemens MAGNETOM magnetic resonance scanner equipped with a local circularly polarized head transmit and a 32 channel brain receive coil (Nova Medical, Inc., Wilmington, Massachusetts). The following parameters were used: 36 axial slices (thickness 1.4 mm, 1.4 × 1.4 mm in‐plane resolution, 224 mm field‐of‐view, anterior‐to‐posterior phase encoding direction) with a 10% inter‐slice gap were recorded in ascending order gradient‐echo, 2 s repetition time, 22 ms echo time, 0.78 ms echo spacing, 1,488 Hz/Px bandwidth, generalized autocalibrating partially parallel acquisition (GRAPPA) with an acceleration factor of 3, 24 Hz/Px bandwidth in phase encoding direction. The field‐of‐view was centered on the approximate location of Heschl's gyrus.

#### 2.1.4 Image pre‐processing

Data pre‐processing was conducted by Hanke et al. (2014). Motion was assessed during the whole session. The median L2‐norm of the estimated translation vector did not exceed 1.8 mm and estimated median L2‐normed rotation did not exceed 1° (Hanke et al., 2014). The raw MRI images were converted from DICOM to NIfTI format, then, a group‐specific template volume for EPI images was calculated in order to aid anatomical alignment across brains. The first volume was extracted as a reference image from each movie segment recording, resulting in 152 images. All images from an individual brain were aligned to its respective reference image from the first movie segment using a rigid body transformation implemented in MCFLIRT30 and averaged to create a template image for each brain. Then, all brains were aligned, by means of an affine transformation using FLIRT30, to the one individual brain with the least root mean square difference to the average image across all brains prior to alignment. The initial alignment target volume was slightly upsampled to 1.2 mm isotropic voxel size to account for spatial oversampling across individuals and movie segments. The alignment target for all subsequent iterations was produced by computing the average image across all aligned brains for each respective iteration. Three more iterations with affine transformations were then followed by 10 nonlinear alignment iterations using FNIRT31 global nonlinear intensity model with bias and 1 cm warp‐field resolution while holding the base affine transformation constant. Lastly, the resulting average image was cropped to the region with maximum overlap across individual brains to create the group EPI template volume. For all subsequent analyses, all data from the 152 movie segments were aligned to this template independently. For each run, the average volume across all time points was computed and aligned to the template through an affine transformation determined by FLIRT while reslicing to 1.2 mm isotropic resolution. In addition, this affine transformation was combined with a nonlinear warping derived by FNIRT and, images were resliced to 1.2 mm isotropic resolution. The entire procedure was implemented in the Nipype framework32 and the source code of the full pipeline is also available at https://openneuro.org/datasets/ds000113/versions/1.3.0.

#### 2.1.5 Functional connectivity and statistical analysis

Statistical analysis was conducted using Python 3.7.3 (Python Software Foundation, http://www.python.org). In Cole's original flexible hub analysis, functional connectivity was contrasted between different task rules (64 in total). Instead of using rule‐division, we divided the movie into small chunks of 30 s (a similar length as used in Cole's analysis). For our design, the choice of window length is somewhat arbitrary, but limited by a number of factors. To achieve good sensitivity to changing connectivity, the window cannot be too short. It should correspond to a timescale for which there were changes in the “implicit task” of the movie. A timescale of tens of seconds is one in which previous work (Zacks, Speer, & Reynolds, 2009) has shown corresponds approximately to single scenes but there will be differences between movies, and it is not clear exactly what chunk length best corresponds to FPN variation. We therefore report results from different chunk lengths in the Appendix S1. We are sharing the code used to compute this analysis (https://github.com/chiaracc/FPNflexiblehubs).

In Experiment 1, for comparability with other studies using the StudyForrest dataset, we used the activation time‐courses provided for 268 ROIs from the Shen atlas (Shen et al., 2013). Subsequent analysis then comprised four stages. The first stage used linear regression analysis to simultaneously estimate context‐independent connectivity, task‐activity, and context‐dependent connectivity for each edge that connects every possible pair of ROIs.

To calculate the regression, we defined a vector to select chunk c:

(1) $$\begin{array}{l} s_{c}\left(t\right)=1\;\text{if}\;d\times c\leq t<d\times \left(c+1\right)\;\text{and}\\ s_{c}\left(t\right)=0\;\text{otherwise} \end{array}$$

where *t* is time in volumes (zero‐based), *d* is chunk duration, *c* is chunk number (zero‐based).

The regression equation was

(2) $$A\left(j,t\right)=\beta^{\left(0\right)}\left(i,j\right)A\left(i,t\right)+\sum_{c=0}^{C-1}\beta_{c}^{\left(1\right)}\left(i,j\right)s_{c}\left(t\right)+\sum_{c=0}^{C-1}\beta_{c}^{\left(2\right)}\left(i,j\right)s_{c}\left(t\right)A\left(i,t\right)+\varepsilon \left(t\right)$$

where A is BOLD activation, ε is the error, j is the target region for which connectivity is being modeled, i the source region, and *C* the total number of chunks. Coefficient $\beta^{\left(0\right)}$ modeled the stationary component of connectivity, coefficients $\beta_{c}^{\left(1\right)}$ the chunk‐specific activity in region j, and coefficients $\beta_{c}^{\left(2\right)}$ the chunk‐specific connectivity. In Stage 1 of our analysis, these coefficients were estimated for each pair of regions by fitting across all timepoints with ordinary least squares.

A chunk size of 30 s (i.e., d=15 volumes*)* was used for the first analysis. Stage 2 of the analysis estimated the extent to which a region's connectivity was flexible and changed across chunks, by calculating the *SD* of the coefficients $\beta_{c}^{\left(2\right)}\left(i,j\right)$ across chunks c for each pair of ROIs $\left(i,j\right)$ and for each participant.

In Stage 3, as in Cole's study (Cole et al., 2013), ROIs were grouped using Power's network division by allocating each ROI of the Shen atlas to the network of the closest region in Power's atlas. We then calculated two different indices for flexibility of the networks: the global network variable connectivity (GVC) and the between‐network variable connectivity (BVC). The GVC is the mean variability in connectivity of each region within a network to every other region in the brain irrespective of whether it was in the same or a different network. In contrast, the BVC only included connections across networks. We calculated both of the indices as Cocuzza, Ito, Schultz, Bassett, and Cole (2020) found that they could give distinct patterns, with regions within the FPN becoming less connected with each other as a function of task.

In Stage 4, ANOVA was used to check if the networks were significantly different from each other, and in particular to test the hypothesis that the frontal parietal network presents bigger changes in connectivity if compared with the other networks.

The full analysis is openly available (https://github.com/chiaracc/FPNflexiblehubs).

The analysis was also repeated for different window lengths (60, 90, 120, 180, and 240 s), and these additional results are reported in the Appendix S1. These window sizes were selected as it has been previously demonstrated that the average correlation values within and between RSNs stabilize at approximately 240 s (van Dijk et al., 2009).

Cocuzza et al. (2020) also calculated connectivity using chunk‐by‐chunk correlation, rather than regression, and found in some cases this provided stronger evidence that the FPN was a flexible hub. Therefore, as an additional analysis we computed the same analysis pipeline, except for Stage 1, that we replaced with a correlation model. The results from this analysis are reported in the Appendix S1.

### 2.2 Results

#### 2.2.1 Global variable connectivity

**FIGURE 1:**
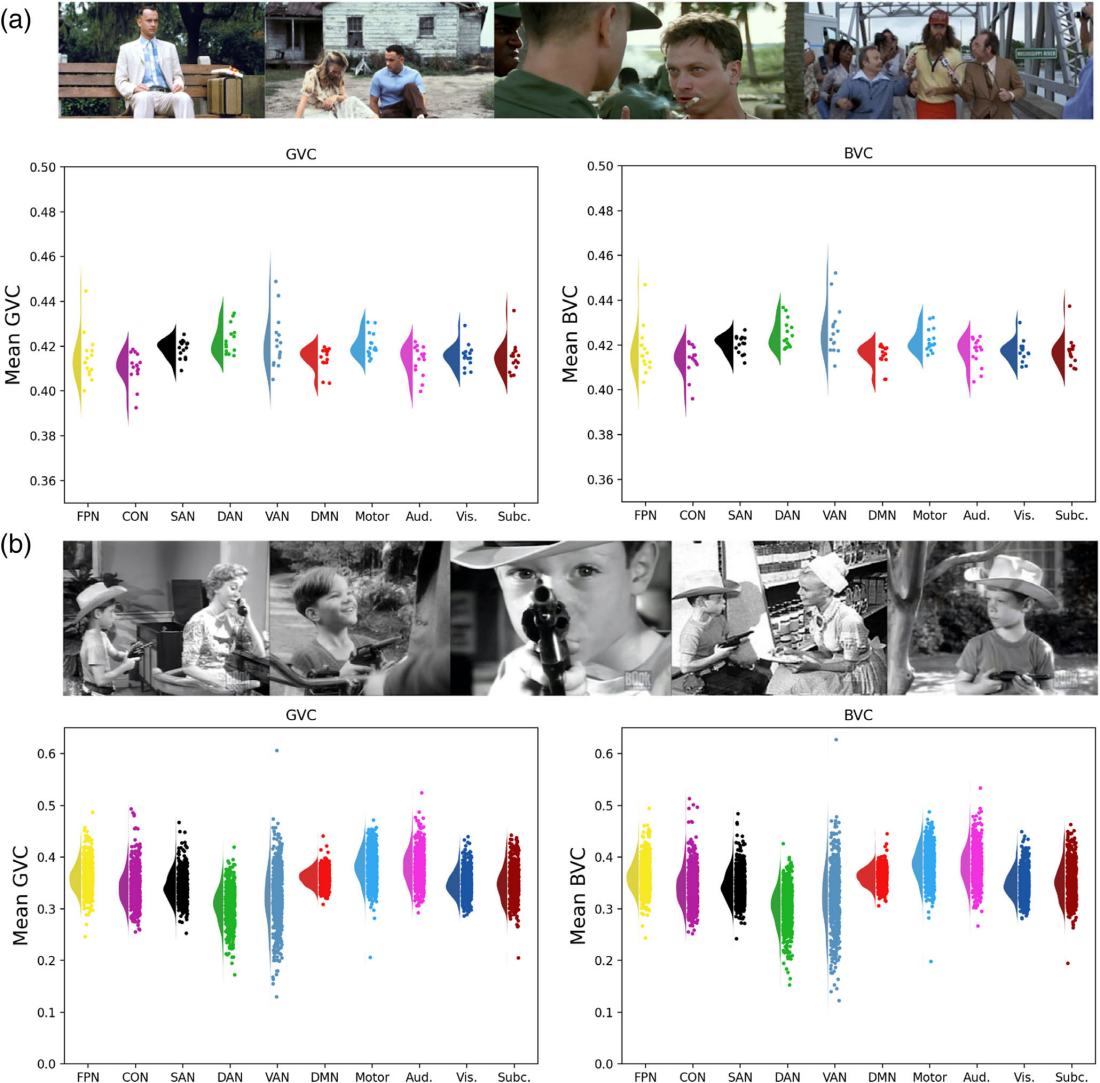
(a,b) The average GVC and BVC across participants for each network are shown: frontal parietal network (FPN), cingulo opercular network (CON), salience network (SAN), dorsal attention network (DAN), ventral attention network (VAN), default mode network (DMN), motor network (Motor), auditory network (Aud.), visual network (Vis.), subcortical network (Subc.), whole brain. Figure 1a shows results from Experiment 1 (Studyforrest) and Figure 1b shows results from Experiment 2 (CamCAN)

**FIGURE 2:**
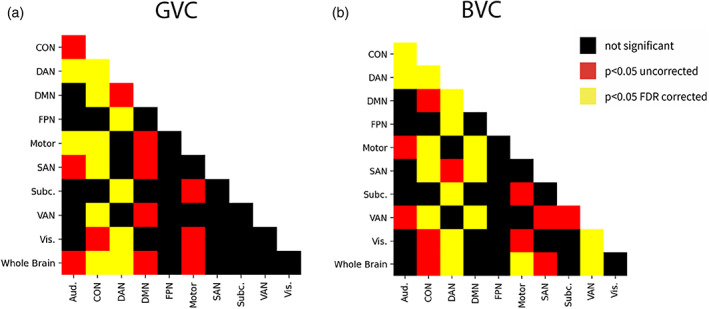
(A,B) All pairwise comparisons between networks are shown, for GVC and for BVC

The mean across subjects for each network shown in Table 1. Figure 1 shows for each network the median, inter quartile range and distribution across subjects of the within‐subject error. A repeated‐measures ANOVA revealed a difference between networks, *F*(10,154) = 4.3964, *p* < .0001. But, the pattern did not support the hypothesis, and in fact in the mean, the FPN had numerically lower flexibility than the average of the brain, or than five other regions (DAN, Motor, SAN, Subc., and Vis). Many of the pairwise differences between networks were significant (Figure 2a), showing that the method does have sufficient sensitivity to detect differences in the flexibility of networks. The FPN showed less flexibility than the DAN (*T* = 3.996, *p* = .016, FDR corrected). But, no significant difference was found between the mean functional connectivity of the whole brain and the FPN. In this analysis, we used a chunk length of 15 volumes (30 s). In the Appendix S1 we report longer chunk lengths (60, 90, 120, 180, and 240 s), which give a similar pattern of results.

**TABLE 1:** GVC and BVC mean and standard deviation across participants for each network is shown for: frontal parietal network (FPN), cingulo opercular network (CON), salience network (SAN), dorsal attention network (DAN), ventral attention network (VAN), default mode network (DMN), motor network (Motor), auditory network (Aud.), visual network (Vis.), subcortical network (Subc.), whole brain (WB)

	Studyforrest		CamCAN	
	GVC	BVC	GVC	BVC
Aud.	0.4137 (0.0137)	0.4187 (0.0092)	0.3830 (0.0569)	0.3838 (0.0584)
CON	0.4102 (0.0138)	0.4133 (0.0097)	0.3420 (0.0568)	0.3437 (0.05863)
DAN	0.4226 (0.0116)	0.4248 (0.0092)	0.3059 (0.0495)	0.3031 (0.0497)
DMN	0.4146 (0.0089)	0.4155 (0.0086)	0.3611 (0.0467)	0.3620 (0.04872)
FPN	0.4149 (0.0143)	0.4174 (0.0118)	0.3608 (0.0543)	0.3633 (0.0558)
Motor	0.4206 (0.099)	0.4221 (0.0088)	0.3819 (0.0575)	0.3876 (0.0567)
SAN	0.4187 (0.0094)	0.4204 (0.0079)	0.3434 (0.0522)	0.3458 (0.0537)
Subc.	0.4151 (0.0117)	0.4172 (0.0096)	0.3481 (0.0571)	0.3525 (0.0754)
VAN	0.4217 (0.0205)	0.4267 (0.0124)	0.3214 (0.0668)	0.3212 (0.0689)
Vis.	0.4157 (0.0009)	0.4174 (0.0081)	0.3492 (0.0506)	0.3498 (0.0528)
Whole brain	0.4176 (0.0068)	0.4177 (0.0071)	0.3565 (0.0440)	0.3570 (0.0461)

#### 2.2.2 Between‐network variable connectivity

This result also generalized to another measure, the BVC, shown in Table 1 and Figure 1. A repeated‐measures ANOVA revealed a difference between networks, *F*(10,154) = 5.5356, *p* < .001. Again, many pairwise comparisons were significant (Figure 2b). The DAN (*T* = 4.3247, FDR corrected *p* = .0208), as well as the Motor (*T* = 3.6332 FDR corrected *p* = .0208) and the VAN (*T* = 2.9535 FDR corrected *p* = .0105) differed from the average *SD* of functional connectivity of the whole brain. No other network differed from the whole brain connectivity or from the FPN, again providing no support that the FPN acts as a flexible hub during naturalistic cognition. The DAN again showed greater flexibility than the FPN (*T* = 3.8669, FDR corrected *p* = .0208).

### 2.3 Discussion

We did not find that the FPN had the highest variability in connectivity to the other networks during naturalistic stimulation. Both our measures of network flexibility [including within‐network connectivity (GVC) or not (BVC)] did not show a difference in the FPN network flexibility, providing no evidence that it acted as a flexible hub during naturalistic stimulation.

The lack of effect for the FPN does not appear to be due to an overall lack of sensitivity as other networks were found to be significantly different from each other. Furthermore, for both the GVC and BVC, the DAN showed greater flexibility than the FPN.

However, there are limitations in the present study. An important difference is that participants were watching a movie that perhaps did not engage the FPN. Perhaps the FPN is still a flexible hub, but only in more intellectually challenging situations than when watching Forrest Gump.

Another technical difference is that in Experiment 1 we used the Shen 268‐region atlas instead of Power 264‐region atlas. As we are summarizing flexibility at the level of whole networks, it is unlikely that the small differences between ROIs led to the reversal of patterns across networks. However, in the next experiment we determined to use the Power 264‐region atlas, which was previously used by Cole and colleagues.

Furthermore, Studyforrest dataset only includes 15 subjects, which is considered to be at the lower end of power for group fMRI analyses (Mumford & Nichols, 2008). For these reasons, we tested the hypothesis on a larger dataset, which has previously been shown to engage the FPN.

## 3 EXPERIMENT 2: CAMBRIDGE CENTRE FOR AGING AND NEUROSCIENCE (CAMCAN)

### 3.1 Methods

#### 3.1.1 CamCAN data

To address the limitations of Experiment 1, in Experiment 2 we analyzed a publicly available fMRI acquisition of 644 pseudo‐anonymous participants (*316 male*, *328 females*; *average age 54.3 years*) watching an 8 min edited clip from the black and white TV episode, “Alfred Hitchcock Presents—Bang! You're Dead.” Participants were asked to simply watch and follow it as best they could. This movie has been previously shown to robustly engage the FPN (Naci et al., 2014). This dataset was collected and made freely available as a part of CamCAN (https://www.cam-can.org/). A detailed description of the dataset is described in Taylor et al. (2017). A proportionate ethical approval was obtained from the ethics committee of the School of Psychology, Trinity College Dublin.

#### 3.1.2 MRI acquisition

MRI images were acquired on a 3T Siemens TIM Trio System at the MRC Cognition Brain and Sciences Unit, Cambridge, United Kingdom. A three‐dimensional (3D) structural MRI was acquired for each participant using T1‐weighted sequence (GRAPPA; repetition time was 2,250 ms; echo time was 2.99 ms; inversion time was 900 ms; flip angle was 9°; matrix size was 256 × 240 × 19 mm; field of view was 256 × 240 × 192 mm; voxel was 1 mm isotropic; a multiband accelerated factor of 2 was used). For the fMRI, T2*‐weighted echo planar images (EPIs) were acquired using a multi‐echo sequence [repetition time was 2.47 s; 5 echoes (echo times were 9.4, 21.2, 33, 45, and 57 ms); flip angle was 78°, 32 axial slices with a thickness of 3.7 mm with an interslice gap of 20%; field of view was 192 × 192 mm; voxel‐size was 3 × 3 × 4.44 mm]; the total acquisition time was 8 min and 13 s.

#### 3.1.3 Image processing

Preprocessing was run using SPM12 (http://www.fil.ion.ucl.ac.uk/spm), as implemented in the automatic analysis (aa; Cusack et al., 2015). The full image processing pipeline is also described in Taylor et al., 2017. Using custom Matlab code, the multiple echoes were combined using the T2* as estimated at each voxel, images were unwarped using the fieldmaps to correct for field inhomogeneities, corrected for subject motion, and slice‐time corrected. Structural images (T1 and T2) were coregistered to a Montreal Neurological Institute (MNI) template image, bias‐corrected, and combined to segment various tissue classes using unified segmentation (Ashburner & Friston, 2005). The segmented gray matter images were then used to create a study specific anatomical template using the DARTEL procedure to optimize interparticipant alignment (Ashburner, 2007), which was then transformed to MNI space. The EPI images were then coregistered to the T1 image and normalized to MNI space using the DARTEL flowfields. The framewise displacement was calculated for each participant, and the average was 0.2409 (*SD* = 0.1909). Head motion was corrected with a wavelet despiking method that removes motion artifacts from the fMRI data without deleting frames from the fMRI time series (Patel et al., 2014, for details see Taylor et al., 2017). The images were finally smoothed with an 8‐mm full width at half maximum (FWHM) Gaussian kernel and the Brain Extraction Tool (Smith, 2002) from the Oxford Centre for Functional Magnetic Resonance Imaging of the Brain's Software Library (FSL version 4.1.8; Smith et al., 2004) was used to remove non brain tissue. Time series normalization was done with a mean‐based intensity normalization (using the 4D grand‐mean) and images were also high‐pass filtered (Gaussian‐weighted least‐squares straight line fitting, equivalent to 100 s) to remove low frequency artifacts and resampled to a resolution of 4 mm. ROI time series extraction was performed using Afni (v.20.2.16) and a 264 ROI parcellation from Power et al. (2011). As in the previous experiment, in this analysis we used a chunk length of 15 volumes (37.05 s). However, in the Appendix S1 we also report longer chunk lengths (74.1, 111.15, 148.2, and 222.3 s).

#### 3.1.4 Functional connectivity and statistical analysis

Statistical analysis was conducted using Python 3.7.3 (Python Software Foundation, http://www.python.org), and it was identical to the one used in Experiment 1 and it is described in Section 2.1.5 except for the use of the Power instead of Shen atlas. The full analysis is openly available (https://github.com/chiaracc/FPNflexiblehubs).

### 3.2 Results

#### 3.2.1 Global variable connectivity

**FIGURE 3:**
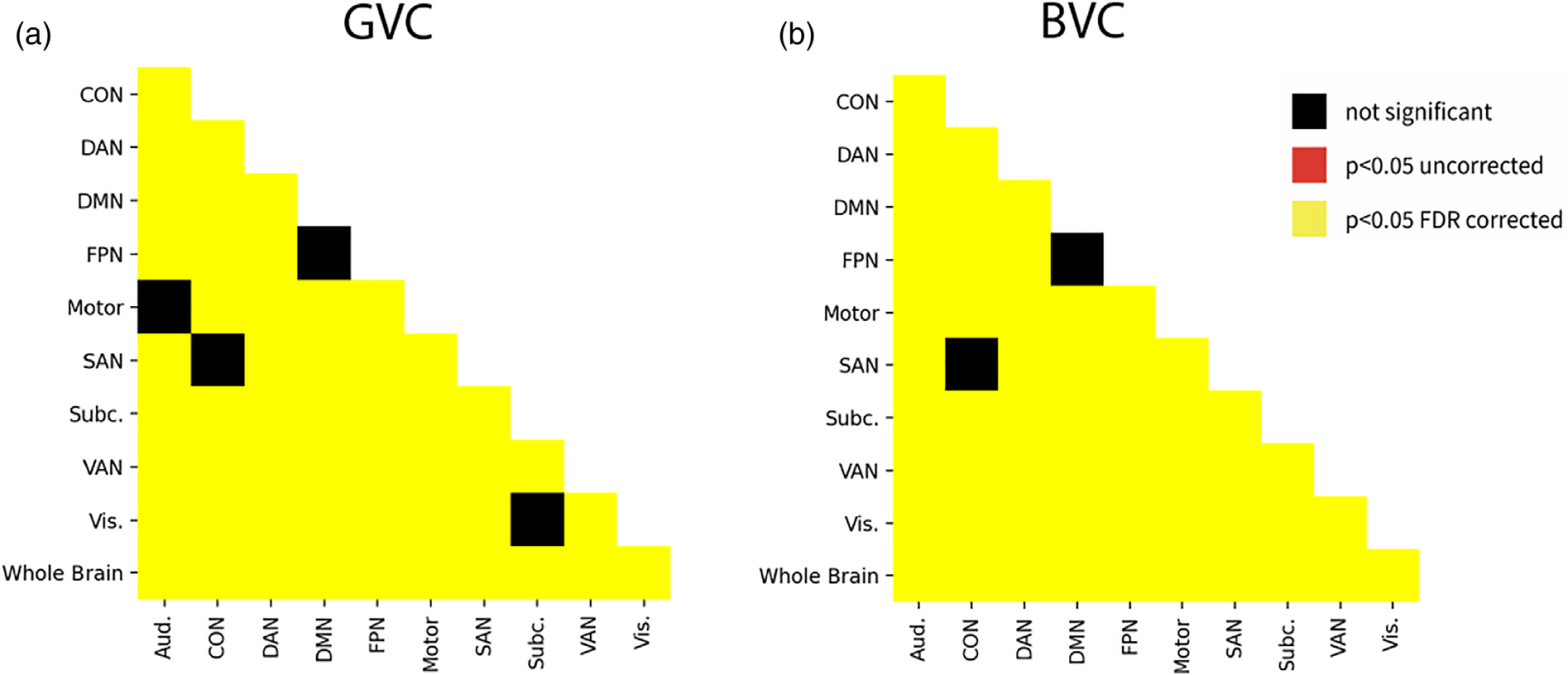
(a,b) All pairwise comparisons between networks are shown, for GVC and for BVC, for the CamCAN dataset

The results are shown in Table 1 and Figure 1. An ANOVA revealed a difference in the degree to which different networks were flexible hubs (*F*[10,7,073] = 215.1633, *p* < .0001). Pairwise comparisons between networks are shown in Figure 3a. The FPN had greater SD of functional connectivity than the average across the brain (*T* = 4.2826, *p* < 0.001 FDR corrected). But, three other networks also showed greater than average: Aud. (*T* = 20.1247, *p* < .001), DMN (*T* = 8.887, *p* < .001), Motor (*T* = 21.8263, *p* < .001). Six networks showed less than average connectivity, CON (*T* = −11.188, *p* < .001), DAN (*T* = −33.6962, *p* < .001), SAN (*T* = −12.2777, *p* < .001), Subc. (*T* = −6.8405, *p* < .001), VAN (*T* = −14.8622, *p* < .001) and Vis. (*T* = −8.8085, *p* < .001). The FPN showed significantly higher flexibility than the CON (*T* = −12.2665, FDR corrected *p* < .001), the DAN (*T* = −26.2231, FDR corrected *p* < .001), and the Motor (*T* = −12.197, FDR corrected *p* < .001); while it showed less flexibility than the Aud. (*T* = 12.1509, FDR corrected *p* < .001), the SAN (*T* = 12.6358, FDR corrected *p* < .001), the Subc. (*T* = 7.3602, FDR corrected *p* < .001), the VAN (*T* = 13.5198, FDR corrected *p* < .001), and the Vis. (*T* = 9.4141, FDR corrected *p* < .001). The detailed statistics of the pairwise comparisons are reported in the Appendix S1.

#### 3.2.2 Between‐network variable connectivity

The results are shown in Table 1 and Figure 1. ANOVA revealed a difference between networks (*F*[10,7,073] = 232.6750, *p* < .0001). Many pairwise comparisons were significant (Figure 3b). The FPN differed from the average SD of functional connectivity of the whole brain (*T* = 6.0174, *p* = .0792) and from Aud. (*T* = 10.5152, *p* < .001 FDR corrected), CON (*T* = −11.9286, *p* < .001), DAN (*T* = −26.72, *p* = .0486), Motor (*T* = −13.1279, *p* < .001), SAN (*T* = 11.9341, *p* < .001), Subc. (*T* = 5.8948, *p* < .001), VAN (*T* = 13.5647, *p* < .001), and Vis. (*T* = 10.264, *p* < .001). The FPN showed higher flexibility than the Aud. (*T* = 10.5152, FDR corrected *p* < .001), SAN (*T* = 11.9341, FDR corrected *p* < .001), Subc. (*T* = 5.8948, FDR corrected *p* < .001), VAN (*T* = 13.5647, FDR corrected *p* < .001), Vis. (*T* = 10.264, FDR corrected *p* < .001), while it showed less flexibility than motor the CON (*T* = −11.9286, FDR corrected *p* < .001), the DAN (*T* = −26.72, FDR corrected *p* < .001), and the Motor (*T* = −13.1279, FDR corrected *p* < .001). All pairwise comparisons are reported in detail in the Appendix S1.

### 3.3 Discussion

Similarly to Experiment 1, and contrary to our hypothesis, with both measures of network flexibility (including within‐network connectivity (GVC) or not (BVC) we did not find the FPN was the network that changed its connectivity the most, providing no evidence that it acted as a flexible hub during naturalistic cognition. Importantly, our method did detect differences in flexibility between networks, with many pairwise comparisons significant. But, the FPN was not the most strongly changing network, again suggesting that a flexible hub behavior is not a trait of this network, but a behavior elicited by a particular type of task, requiring quick rule shifts from one rule to another. The short clip (~10 min) that participants viewed in Experiment 2 was shown to activate the FPN in healthy adults and vegetative patients (Naci et al., 2014), therefore, differently from Experiment 1, here the FPN should be engaged enough. Again, the “flexible hub” behavior seems not to be a trait that is universally shown by the FPN.

## 4 GENERAL DISCUSSION

In previous work, when performing a set of demanding tasks, the FPN was found to show greater dynamic variation in functional connectivity than other brain networks (Cocuzza et al., 2020; Cole et al., 2013). The hypothesis tested here is that this will generalize, and the FPN will also show greater dynamic variation in functional connectivity during naturalistic cognition. The results do not support the hypothesis. We calculated the functional connectivity of a set of brain networks using two freely available datasets of people watching a movie. We used a similar pipeline to the one used in Cole et al. (2013), and in Experiment 1 we used data from a freely available dataset of 15 people watching an entire movie (“Forrest Gump”). In Experiment 1, the DAN was the only network with significantly more flexible connectivity. In Experiment 2, we analyzed data from a bigger sample (*N* = 644) watching a short and engaging clip by Alfred Hitchcock. Again, we found that the FPN's connectivity pattern was not the most flexible.

In Cole's original study, participants performed a set of tasks, the timing of which was determined by the experimenter. Functional connectivity was then compared between tasks. A challenge with imaging paradigms for which there is no experimenter‐defined timing, such as resting‐state fMRI, or the naturalistic stimulation used in our two experiments, is to know on what time scale to examine changes in dynamic connectivity. In dynamic functional connectivity experiments, window lengths of 30–240 are typically used (Hutchison et al., 2013). A previous study using movies to study brain connectivity showed how longer time courses preserved individual characteristics in connectivity profiles (Finn et al., 2015). We had to choose a tradeoff between a window that is too long and therefore not sensitive to flexibility, and too short and thus insufficiently statistically powerful enough to estimate functional connectivity. We, therefore, conducted analyses that spanned a broad range, with windows from 30 to 240 s. This method was successful, in that many pairwise differences were found in the variability between different networks. However, across the range of time windows, in neither experiment was the FPN ever found to show the greatest variation in connectivity.

Two metrics have been used to assess the flexibility of the FPN (Cocuzza et al., 2020; Cole et al., 2013). The GVC quantifies the degree to which each region in each network changes its connectivity with all of the other regions, irrespective of whether they are in the same network or a different one. The BVC, in contrast, only examines between‐network connections. These two different measures have been found previously to show quite different patterns (Cocuzza et al., 2020; Cole et al., 2013). We, therefore, evaluated both metrics at each window length, and again found that the FPN never had the greatest variation in connectivity. It is worth noting that the absolute values of GVC and BVC will be affected by many factors, including the signal‐to‐noise, which will be affected by the MRI machine and scanning protocol. Comparison of the absolute values of the current study and previous studies is not therefore informative.

Why might the FPN not have been the most flexible network in the context of the movies? It might be that these movies do not engage the FPN enough, especially if compared with the highly demanding frontal tasks used in Cole et al. (2013). However, previous studies showed how movies engage a broad range of brain networks (Spiers & Maguire, 2007; Vanderwal, Kelly, Eilbott, Mayes, & Castellanos, 2015). Furthermore, the clip used in the second experiment was found to elicit FPN activation in a previous study (Naci et al., 2014). There is no reason to believe that the FPN activity evoked by these movies is particularly lower than that evoked in everyday life. In contrast, Cole's tasks were designed to be taxing and evoke the strongest possible FPN activity. Therefore, the brain state we are measuring is more representative of everyday cognition.

Our results do not imply that connectivity of the FPN, or changes in its pattern of connectivity, are not important for cognition. The FPN is consistently engaged by demanding cognition (Cole et al., 2013; Duncan et al., 2000; Kievit et al., 2014). These areas are not domain specific to a particular type of input or output, and they engage with different brain networks across a variety of tasks. They are, therefore, anatomically highly connected to the rest of the brain, and receive input from or to control more specialized areas of the brain (Duncan et al., 2000; Spreng et al., 2013). It is not clear what aspect of Cole's tasks is critical. The FPN may be engaged and act as a flexible hub only when tasks are sufficiently difficult. Alternatively, perhaps the variety in Cole's tasks is important, with some tasks more cognitively and some more motorically challenging. Or, it could be the relative abstract nature of the tasks, with simple stimuli and precise well‐defined instructions, that leads to sharply differing connectivity requirements.

An alternative explanation is that it is not the level of engagement of the FPN that differs between Cole's taxing tasks and naturalistic movie viewing, but rather than engagement of other brain networks. Our results show that broad connectivity is not unique to the FPN. Even “unimodal” regions are connected to many other brain regions, and in the context of complex naturalistic stimulation, may show flexible dynamic connectivity, such as in Experiment 2, where the auditory and motor networks acted even more strongly as flexible hubs than the DMN or FPN. Psychological tasks designed to probe “frontal function” such as those used by Cole attempt to maximize the engagement of the frontal lobe through the imposition of sets of complex abstract rules, while minimizing the demand on other brain systems. They do not, for example, involve perceptually challenging discriminations, or movements that are in themselves challenging to perform. In contrast, naturalistic situations are richer and typically challenging in a broader range of ways, and watching a movie could be considered a more “bottom‐up” mode, in which no decisions or motor outputs are required; movie‐viewing also elicits a wider range of emotions, and may require more frequent memory retrieval and more frequent and greater surprises.

At a broader scale, our results present a further challenge for the simple mapping between psychological processes and brain regions. While it is conceptually more straightforward to imagine a separation between “unimodal” regions and more flexible amodal regions, neuroscience has shown that many brain connections are recurrent—with even V1 receiving more descending than ascending connections. The “global workspace” in which information from different modalities is brought together (Dehaene et al., 1998) likely does not correspond to a specific brain region like the FPN, but is rather a dynamically configured network of regions, coordinated for example by the synchrony of oscillations (Grossberg, 2013). The insight from the current study is that to characterize this, it may be important to measure brain function in the context of naturalistic stimulation, rather than with tasks that have been carefully optimized to isolate one component of the system.

## CONFLICT OF INTEREST

The authors declare no conflicts of interest.

## ETHICS STATEMENT

Ethical approval was obtained from the ethics committee of the School of Psychology, Trinity College Dublin.

Participant consent can be found at http://www.cam-can.com/index.php?content=datause.

## Supporting information


**Appendix S1** Supporting Information

## Data Availability

The data that support the findings of this study were derived from the following resources available in the public domain: www.studyforrest.org and www.cam-can.org.
