# Supplementary material for: The fronto‐parietal network is not a flexible hub during naturalistic cognition
Source: Hum Brain Mapp. 2021 Oct 15;43(2):750–9. doi: 10.1002/hbm.25684 (PMC8720185; doi:10.1002/hbm.25684)
Supplement: Supplementary file 1 — Appendix S1 Supporting Information [file HBM-43-750-s001.pdf]

Supplementary Materials

1. StudyForrest additional results

1.1 Network pairwise comparisons

Table 1

All the pairwise comparisons for all the networks are shown, for GVC and BVC for the StudyForrest dataset.

Studyforrest							
GVC				BVC			
Networks		T	FDR corrected p value	Networks		T	FDR corrected p value
Aud.	CON	2.4057	NS	Aud.	CON	2.8032	p<0.05
Aud.	DAN	-3.0074	p<0.05	Aud.	DAN	-2.7679	p<0.05
Aud.	DMN	-0.7814	NS	Aud.	DMN	1.3759	NS
Aud.	FPN	-0.3033	NS	Aud.	FPN	-0.1487	NS
Aud.	Motor	-2.9904	p<0.05	Aud.	Motor	-2.3816	NS
Aud.	SAN	-2.4825	NS	Aud.	SAN	-1.8704	NS
Aud.	Subc.	-0.5633	NS	Aud.	Subc.	-0.1397	NS
Aud.	VAN	-2.0648	NS	Aud.	VAN	-2.6653	NS
Aud.	Vis.	-0.8144	NS	Aud.	Vis.	-0.2387	NS
Aud.	Whole Brain	-2.3881	NS	Aud.	Whole Brain	-0.5271	NS
CON	DAN	-3.9493	p<0.05	CON	DAN	-3.7992	p<0.05
CON	DMN	-4.8168	p<0.05	CON	DMN	-2.467	NS
CON	FPN	-1.1355	NS	CON	FPN	-1.0182	NS
CON	Motor	-3.9623	p<0.05	CON	Motor	-3.4912	p<0.05
CON	SAN	-3.7261	NS	CON	SAN	-3.2515	p<0.05

CON	Subc.	-1.6187	NS	CON	Subc.	-1.356	NS
CON	VAN	-3.1284	NS	CON	VAN	-3.7915	p<0.05
CON	Vis.	-2.8012	NS	CON	Vis.	-2.1781	NS
CON	Whole Brain	-4.1549	p<0.05	CON	Whole Brain	-2.5316	NS
DAN	DMN	2.8574	NS	DAN	DMN	3.4664	p<0.05
DAN	FPN	3.996	p<0.05	DAN	FPN	3.8669	p<0.05
DAN	Motor	1.012	NS	DAN	Motor	1.3596	NS
DAN	SAN	1.811	NS	DAN	SAN	2.1582	NS
DAN	Subc.	3.3421	p<0.05	DAN	Subc.	3.5158	p<0.05
DAN	VAN	0.222	NS	DAN	VAN	-0.5215	NS
DAN	Vis.	3.3574	p<0.05	DAN	Vis.	3.7983	p<0.05
DAN	Whole Brain	2.9534	p<0.05	DAN	Whole Brain	4.3247	p<0.05
DMN	FPN	-0.0791	NS	DMN	FPN	-0.533	NS
DMN	Motor	-2.7676	NS	DMN	Motor	-3.2236	p<0.05
DMN	SAN	-2.3676	NS	DMN	SAN	-2.9675	p<0.05
DMN	Subc.	-0.1816	NS	DMN	Subc.	-0.684	NS
DMN	VAN	-2.2167	NS	DMN	VAN	-3.6698	p<0.05
DMN	Vis.	-0.6163	NS	DMN	Vis.	-1.1788	NS
DMN	Whole Brain	-2.3969	NS	DMN	Whole Brain	-1.838	NS
FPN	Motor	-1.7831	NS	FPN	Motor	-1.5096	NS
FPN	SAN	-1.249	NS	FPN	SAN	-0.9702	NS
FPN	Subc.	-0.0724	NS	FPN	Subc.	0.0997	NS
FPN	VAN	-1.4574	NS	FPN	VAN	-2.056	NS
FPN	Vis.	-0.2525	NS	FPN	Vis.	0.0156	NS
FPN	Whole Brain	-0.9762	NS	FPN	Whole Brain	-0.0857	NS
Motor	SAN	1.0132	NS	Motor	SAN	1.0431	NS
Motor	Subc.	2.729	NS	Motor	Subc.	2.5134	NS
Motor	VAN	-0.3262	NS	Motor	VAN	-1.3414	NS
Motor	Vis.	2.4025	NS	Motor	Vis.	2.455	NS
Motor	Whole Brain	2.3088	NS	Motor	Whole Brain	3.6332	p<0.05
SAN	Subc.	1.5951	NS	SAN	Subc.	1.4421	NS
SAN	VAN	-0.9614	NS	SAN	VAN	-2.1483	NS

SAN	Vis.	1.849	NS	SAN	Vis.	1.9381	NS
SAN	Whole Brain	0.9689	NS	SAN	Whole Brain	2.5039	NS
Subc.	VAN	-1.6498	NS	Subc.	VAN	-2.4853	NS
Subc.	Vis.	-0.2012	NS	Subc.	Vis.	-0.0704	NS
Subc.	Whole Brain	-1.3189	NS	Subc.	Whole Brain	-0.2603	NS
VAN	Vis.	1.7325	NS	VAN	Vis.	2.8498	p<0.05
VAN	Whole Brain	1.2709	NS	VAN	Whole Brain	2.9535	p<0.05
Vis.	Whole Brain	-1.5779	NS	Vis.	Whole Brain	-0.2438	NS

1.2 Between-Network Variable Connectivity with different chunklength

Results are shown in Table 2.
For chunks of 60 seconds, ANOVA revealed a difference between networks, $F(10,154)=4.5784$, $p<0.001$. Tukey's honest significance test (Tukey, 1949) showed no difference between the mean functional connectivity of the whole brain and any particular network, however the Vis. differed from the DMN ($T=5.3669$, corrected $p<0.01$).
For chunks of 90 seconds, ANOVA revealed a difference between networks, $F(10,154)=5.0382$, $p<0.001$. Tukey's honest significance test revealed that the DAN ($T=4.1624$, corrected $p<0.05$), Motor ($T=3.2714$, correctedp<0.001), and Vis. ($T=-4.7813$, corrected $p<0.05$) differed from the average SD of functional connectivity of the whole brain.
For chunks of 120 seconds, ANOVA revealed a difference between networks, $F(10,154)=5.1523$, $p<0.001$. Tukey's honest significance test (Tukey, 1949) revealed that only the Vis. ($T=-5.9324$, corrected $p<0.01$), differed from the average SD of functional connectivity of the whole brain.
For chunks of 180 seconds, ANOVA revealed a difference between networks, $F(10,154)=6.6087$, $p<0.001$. Tukey's honest significance test revealed that only the Vis. ($T=-5.6945$, corrected $p=0.0001$) and Vis. ($T=-5.6945$, corrected $p<0.01$) differed from the average SD of functional connectivity of the whole brain.
For chunks of 240 seconds, ANOVA revealed a difference between networks, $F(10,154)=6.3554$, $p<0.001$. Tukey's honest significance test revealed that DMN ($T=4.32474.2137$, corrected $p<0.05$) and the Vis. ($T=-6.0314$, corrected $p<0.01$) differed from the average SD of functional connectivity of the whole brain.

1.3 Global Variable Connectivity with different chunklength

Results are shown in Table 2. For chunks of 60 seconds, ANOVA revealed a difference between networks, $F(10,154)=3.7493915997073$, $p<0.001$. Tukey's honest significance test revealed that the Vis. ($T=-4.2212$, corrected $p<0.05$) differed from the average SD of functional connectivity of the whole brain).

For chunks of 90 seconds, ANOVA revealed a difference between networks, $F(10,154)=4.2376$, $p<0.001$. Tukey's honest significance test revealed that the DAN ($T=4.1624$, corrected $p<0.05$), and Vis. ($T=-4.7813$, corrected $p<0.05$) differed from the average SD of functional connectivity of the whole brain.

For chunks of 120 seconds, ANOVA revealed a difference between networks, $F(10,154)=4.3693$, $p<0.001$. Tukey's honest significance test revealed that only the Vis. ($T=-6.5945$, corrected $p<0.001$), differed from the average SD of functional connectivity of the whole brain.

For chunks of 180 seconds, ANOVA revealed a difference between networks, $F(10,154)=6.0197$, $p<0.001$. Tukey's honest significance test revealed that only the Vis. ($T=-5.6945$, corrected $p<0.01$), differed from the average SD of functional connectivity of the whole brain.

For chunks of 240 seconds, ANOVA revealed a difference between networks, $F(10,154)=5.8337208860$, $p<0.001$. Tukey's honest significance test revealed that DMN ($T=4.325$, corrected $p<0.05$) and the Vis. ($T=--6.0639$, corrected $p<0.01$) differed from the average SD of functional connectivity of the whole brain.

Table 2
The average standard deviation across participants for each network for each chunk length calculated with a regression model is shown for: Frontal Parietal Network (FPN), Cingulo Opercular Network (CON), Salience Network (SAN), Dorsal Attention Network (DAN), Ventral Attention Network (VAN), Default Mode Network (DMN), Motor Network (Motor), Auditory Network (Aud.), Visual Network (Vis.), Subcortical Network (Subc.), Whole Brain (WB).

Studyforrest										
	GVC					BVC				
	60 s	90 s	120 s	180 s	240 s	60 s	90 s	120 s	180 s	240 s
Aud.	0.3101	0.2644	0.2379	0.2062	0.1894	0.3125	0.2666	0.2398	0.2078	0.1907
CON	0.3071	0.2611	0.2343	0.2023	0.1861	0.3094	0.2630	0.2360	0.2036	0.1872
DAN	0.3147	0.2669	0.2391	0.2058	0.1883	0.3164	0.2683	0.2403	0.2068	0.1893
DMN	0.3109	0.2649	0.2377	0.2051	0.1882	0.3114	0.2653	0.2379	0.2052	0.1882
FPN	0.3091	0.2618	0.2336	0.1998	0.1828	0.3112	0.2637	0.235	0.2012	0.1841
Motor	0.3133	0.2656	0.2374	0.2033	0.1860	0.3145	0.2665	0.2383	0.2041	0.1867
SAN	0.3118	0.2646	0.2370	0.2039	0.1868	0.3169	0.2656	0.2380	0.2049	0.1877
Subc.	0.3105	0.2638	0.2362	0.2033	0.1860	0.3122	0.2652	0.2375	0.2044	0.1870
VAN	0.3129	0.2644	0.2358	0.2006	0.1832	0.3162	0.2672	0.2385	0.2030	0.1857
Vis.	0.3084	0.2605	0.2326	0.1988	0.1816	0.3098	0.2617	0.2336	0.1996	0.1823
Whole Brain	0.3116	0.2644	0.2368	0.2034	0.18631	0.3117	0.2645	0.2368	0.2034	0.1863

1.4 Correlation results of Global-Network Variable Connectivity

For chunks of 30 seconds, ANOVA revealed a difference between networks, F(10,154)=6.9142, p<0.001. Tukey's honest significance test revealed that the Vis. (T=4.3853 , corrected p=0.0336) differed from the average SD of functional connectivity of the whole brain).

For chunks of 60 seconds, ANOVA revealed a difference between networks, F(10,154)=4.5697, p<0.001. Tukey's honest significance test revealed no difference between any networks and the average SD of functional connectivity of the whole brain. However, Vis. differed from the CON (T=4.3853, corrected p<0.05) and DMN (T=-4.6286, corrected p<0.05).

For chunks of 90 seconds, ANOVA revealed a difference between networks, F(10,154)=8.0205, p<0.001. Tukey's honest significance test revealed that FPN (T=-5.6727, corrected p<0.01), CON (T=-4.7952, corrected p<0.05) and Motor (T=-4.3808, corrected p<0.05) differed from the average SD of functional connectivity of the whole brain.

For chunks of 120 seconds, ANOVA revealed a difference between networks, F(10,154)=12.6269, p<0.001. Tukey's honest significance test revealed that the Fronto-Parietal Network (FPN; T=-6.4448, corrected p<0.001), the Aud. (T=-4.6774, corrected p<0.05) the CON (T=-5.3908, corrected p<0.01) and the Motor (T=-5.1916, corrected p<0.01) differed from the average SD of functional connectivity of the whole brain.

For chunks of 180 seconds, ANOVA revealed a difference between networks (F(10,154)=18.21328376477, p<0.001). Tukey's honest significance test revealed that the FPN (T=-6.5248, corrected p<0.001), the Aud. (T=-5.5673, corrected p<0.01) the CON (T=-6.5365, corrected p<0.001) and Motor (T=-6.6628, corrected p<0.001) differed from the average SD of functional connectivity of the whole brain.

For chunks of 240 seconds, ANOVA revealed a difference between networks, F(10,154)=22.0223, p<0.001. Tukey's honest significance test revealed that the Fronto-Parietal Network (FPN; T=-7.0644, corrected p<0.001), Aud. (T=-6.0583, corrected p<0.01) =, CON (T=-6.0895, corrected p<0.005) and the Motor (T=-6.7869, corrected p<0.005) differed from the average SD of functional connectivity of the whole brain.

All results are available at https://github.com/chiaracc/FPNflexiblehubs/tree/main/res/studyforrest.

1.5 Correlation results of Between-Network Variable Connectivity

For chunks of 30 seconds, ANOVA revealed a difference between networks, $F(10,154)=8.972169502473$, $p<0.001$. Tukey's honest significance test revealed that the Vis. ($T=4.3853$, corrected $p=0.0336$) differed from the average SD of functional connectivity of the whole brain.

For chunks of 60 seconds, ANOVA revealed a difference between networks, $F(10,154)=5.47247244126$, $p<0.001$. Tukey's honest significance test revealed no difference between any networks and the average SD of functional connectivity of the whole brain. However, Vis. different from CON ($T=4.3853$, corrected $p<0.05$) and DMN ($T=-4.6286$, corrected $p<0.05$).

For chunks of 90 seconds, ANOVA revealed a difference between networks, $F(10,154)=6.826611258$, $p<0.001$. Tukey's honest significance test revealed that FPN ($T=-4.2343$, corrected $p<0.05$) and CON ($T=-4.9022$, corrected $p<0.05$) differed from the average SD of functional connectivity of the whole brain.

For chunks of 120 seconds, ANOVA revealed a difference between networks, $F(10,154)=10.858254993$, $p<0.001$. Tukey's honest significance test revealed that FPN ($T=-6.1039$, corrected $p<0.005$), and CON ($T=-4.7892$, corrected $p=0.0138$) and the Motor ($T=-4.068$, corrected $p<0.05$) differed from the average SD of functional connectivity of the whole brain.

For chunks of 180 seconds, ANOVA revealed a difference between networks ($F(10,154)=16.0999$, $p<0.001$). Tukey's honest significance test revealed that FP ($T=-6.3512$, corrected $p<0.001$), CON ($T=-5.5699$, corrected $p<0.005$), Motor ($T=-5.5379$, corrected $p<0.005$) differed from the average SD of functional connectivity of the whole brain.

For chunks of 240 seconds, ANOVA revealed a difference between networks, $F(10,154)=19.7073$, $p<0.001$. Tukey's honest significance test revealed that FPN ($T=-6.7336$, corrected $p<0.001$), Aud. ($T=-5.7073$, corrected $p=<0.01$), CON ($T=-5.21$, corrected $p<0.01$) and Motor ($T=-5.7882$, corrected $p<0.005$) differed from the average SD of functional connectivity of the whole brain.

All results are available at https://github.com/chiaracc/FPNflexiblehubs/tree/main/res/studyforrest.

2. CamCAN additional results

2.1 Network pairwise comparisons

Table 3
All the pairwise comparisons for all the networks are shown.

CamCAN							
GVC				BVC			
Networks		T	FDR corrected p value	Networks		T	FDR corrected p value
Aud.	CON	19.8791	p<0.001	Aud.	CON	17.7626	p<0.001
Aud.	DAN	43.6624	p<0.001	Aud.	DAN	42.405	p<0.001
Aud.	DMN	15.7214	p<0.001	Aud.	DMN	14.3903	p<0.001
Aud.	FPN	12.1509	p<0.001	Aud.	FPN	10.5152	p<0.001
Aud.	Motor	0.5341	NS	Aud.	Motor	-2.0019	NS
Aud.	SAN	21.1705	p<0.001	Aud.	SAN	18.7259	p<0.001
Aud.	Subc.	17.6938	p<0.001	Aud.	Subc.	14.5993	p<0.001
Aud.	VAN	25.6609	p<0.001	Aud.	VAN	24.3494	p<0.001
Aud.	Vis.	19.1617	p<0.001	Aud.	Vis.	17.7389	p<0.001
Aud.	Whole Brain	20.1247	p<0.001	Aud.	Whole Brain	18.6793	p<0.001
CON	DAN	15.7451	p<0.001	CON	DAN	16.2222	p<0.001
CON	DMN	-12.766	p<0.001	CON	DMN	-11.281	p<0.001
CON	FPN	-12.2665	p<0.001	CON	FPN	-11.9286	p<0.001
CON	Motor	-21.1276	p<0.001	CON	Motor	-21.4497	p<0.001
CON	SAN	-1.0098	NS	CON	SAN	-1.423	NS
CON	Subc.	-3.4787	p<0.001	CON	Subc.	-4.6461	p<0.001
CON	VAN	6.7364	p<0.001	CON	VAN	6.7986	p<0.001
CON	Vis.	-4.8486	p<0.001	CON	Vis.	-3.8059	p<0.001
CON	Whole Brain	-11.188	p<0.001	CON	Whole Brain	-9.478	p<0.001
DAN	DMN	-34.7273	p<0.001	DAN	DMN	-33.9321	p<0.001
DAN	FPN	-26.2231	p<0.001	DAN	FPN	-26.72	p<0.001

DAN	Motor	-40.2762	p<0.001	DAN	Motor	-41.0157	p<0.001
DAN	SAN	-19.4252	p<0.001	DAN	SAN	-20.1904	p<0.001
DAN	Subc.	-19.701	p<0.001	DAN	Subc.	-21.0196	p<0.001
DAN	VAN	-8.5084	p<0.001	DAN	VAN	-9.2967	p<0.001
DAN	Vis.	-21.5575	p<0.001	DAN	Vis.	-21.2493	p<0.001
DAN	Whole Brain	-33.6962	p<0.001	DAN	Whole Brain	-32.6852	p<0.001
DMN	FPN	0.2915	NS	DMN	FPN	-1.0548	p>0.05
DMN	Motor	-13.9104	p<0.001	DMN	Motor	-15.8452	p<0.001
DMN	SAN	14.4461	p<0.001	DMN	SAN	12.2446	p<0.001
DMN	Subc.	9.4994	p<0.001	DMN	Subc.	6.4842	p<0.001
DMN	VAN	16.0694	p<0.001	DMN	VAN	15.384	p<0.001
DMN	Vis.	11.9304	p<0.001	DMN	Vis.	11.3803	p<0.001
DMN	Whole Brain	8.887	p<0.001	DMN	Whole Brain	9.0424	p<0.001
FPN	Motor	-12.197	p<0.001	FPN	Motor	-13.1279	p<0.001
FPN	SAN	12.6358	p<0.001	FPN	SAN	11.9341	p<0.001
FPN	Subc.	7.3602	p<0.001	FPN	Subc.	5.8948	p<0.001
FPN	VAN	13.5198	p<0.001	FPN	VAN	13.5647	p<0.001
FPN	Vis.	9.4141	p<0.001	FPN	Vis.	10.264	p<0.001
FPN	Whole Brain	4.2826	p<0.001	FPN	Whole Brain	6.0174	p<0.001
Motor	SAN	21.0415	p<0.001	Motor	SAN	21.1409	p<0.001
Motor	Subc.	18.9096	p<0.001	Motor	Subc.	18.2179	p<0.001
Motor	VAN	24.4072	p<0.001	Motor	VAN	24.9915	p<0.001
Motor	Vis.	20.0906	p<0.001	Motor	Vis.	21.4579	p<0.001
Motor	Whole Brain	21.8263	p<0.001	Motor	Whole Brain	24.2367	p<0.001
SAN	Subc.	-2.8179	NS	SAN	Subc.	-3.6985	p<0.001
SAN	VAN	8.0535	p<0.001	SAN	VAN	8.3057	p<0.001
SAN	Vis.	-4.1197	p<0.001	SAN	Vis.	-2.6328	p<0.01
SAN	Whole Brain	-12.2777	p<0.001	SAN	Whole Brain	-9.7236	p<0.001
Subc.	VAN	8.9861	p<0.001	Subc.	VAN	9.7184	p<0.001
Subc.	Vis.	-0.9059	NS	Subc.	Vis.	2.2046	p<0.05
Subc.	Whole Brain	-6.8405	p<0.001	Subc.	Whole Brain	-3.425	p<0.001
VAN	Vis.	-9.8251	p<0.001	VAN	Vis.	-9.369	p<0.001

VAN	Whole Brain	-14.8622	p<0.001	VAN	Whole Brain	-14.0571	p<0.001
Vis.	Whole Brain	-8.8085	p<0.001	Vis.	Whole Brain	-8.0872	p<0.001

2.2 Global-Network Variable Connectivity with different chunklength

Results are shown in Table 4.
For chunks of 60 seconds, ANOVA revealed a difference between networks, F(10,7073)=305.4096, p<0.001. Tukey's honest significance test revealed that FPN (T=6.8219, corrected p=0.0425), the Aud. (T=20.2601, corrected p<0.0001), CON (T=-10.8871, p<0.001), DAN (T=-31.7311, p<0.001), DMN (T=6.071, p<0.001), Motor (T=21.6498, p<0.001), SAN (-10.0294, p<0.001), VAN (T=-11.9288, p<0.001) and Vis. (T=-12.0198, p<0.001) differed from the average SD of functional connectivity of the whole brain.
For chunks of 90 seconds, ANOVA revealed a difference between networks, F(10,7073)=242.7420, p<0.001. Tukey's honest significance test revealed that FPN (T=10.1151, corrected p<0.0001), Aud. (T=15.0691, corrected p<0.0001), CON (T=-9.3208, corrected p<0.001), DAN (T=-27.8893, corrected p<0.001), DMN (T=4.6597, corrected p<0.001), Motor (T=22.6782, corrected p<0.001), SAN (T=-7.563, corrected p<0.001), Subc. (T=-9.4377, corrected p<0.0001), VAN (T=-9.6279, corrected p<0.001) and Vis. (T=-10.351, corrected p<0.001) differed from the average SD of functional connectivity of the whole brain.
For chunks of 120 seconds, ANOVA revealed a difference between networks F(10,7073)=229.43925, p<0.001. Tukey's honest significance test revealed that FPN (T=7.4594, corrected p<0.0001), Aud. (T=18.6344, corrected p=0.0024), CON (T=-9.1369, corrected p<0.001), DAN (T=-26.6773, corrected p<0.001), DMN (T=5.2968, corrected p<0.001), Motor (T=18.835, corrected p<0.0001), SAN (T=-7.7123, corrected p<0.001), Subc. (T=-13.73, corrected p<0.0001), VAN (T=-7.6765, corrected p<0.001) and Vis. (T=-14.4248, corrected p<0.001) differed from the average SD of functional connectivity of the whole brain.
For chunks of 180 seconds, ANOVA revealed a difference between networks (F(10,7073)=175.4594, p<0.001). Tukey's honest significance test revealed that FPN (T=6.902, corrected p<0.0001), Aud. (T=15.1187, corrected p<0.0001), CON (T=-11.0445, corrected p<0.001), DMN (T=3.7391, corrected p<0.001), Motor (T=17.3546, corrected

p<0.0001), SAN (T=-5.7782, corrected p<0.001), Subc. (T=-14.9502, corrected p<0.0001), VAN (T=-3.9464, corrected p<0.001) and Vis. (T=-12.9977, corrected p<0.001) differed from the average SD of functional connectivity of the whole brain.
Results from chunks of 240 s are not shown as the movie clip used in CamCAN was too short.

2.3 Between-Network Variable Connectivity with different chunklength

Results are shown in Table 4.
For chunks of 60 seconds, ANOVA revealed a difference between networks F(10,7073)=305.3112, p<0.001. Tukey's honest significance test revealed that FPN (T=8.876, corrected p<0.0001), Aud. (T=17.9568, corrected p<0.01), CON (T=-9.2692, p<0.001), DAN (T=-31.6175, p<0.001), DMN (T=5.6407, p<0.001), Motor (T=24.2182, p<0.001), SAN (T=-7.8514, p<0.001), Subc. (T=-6.3571, corrected p<0.0001), VAN (T=-11.5051, p<0.001) and Vis. (T=-11.3513, p<0.001) differed from the average SD of functional connectivity of the whole brain.
For chunks of 90 seconds, ANOVA revealed a difference between networks F(10,7073)=249.9604, p<0.001. Tukey's honest significance test revealed that FPN (T=10.1151, corrected p<0.0001), Aud. (T=15.0691, corrected p=0<0.0001), CON (T=-9.3208, corrected p=0<0.0001), DAN (T=-27.889, corrected p<0.001), DMN (T=4.6597, corrected p<0.001), Motor (T=22.6782, corrected p=0<0.0001), SAN (T=-7.5638, corrected p<0.001), Subc. (T=-9.4377, corrected p<0.0001), VAN (T=-9.6279, corrected p<0.001) and Vis. (T=-10.351, corrected p<0.0001) differed from the average SD of functional connectivity of the whole brain.
For chunks of 120 seconds, ANOVA revealed a difference between networks F(10,7073)=238.2426, p<0.001. Tukey's honest significance test revealed that FPN (T=9.2507, corrected p<0.0001), Aud. (T=14.901, corrected p<0.0001), CON (T=-7.3253, corrected p<0.001), DAN (T=-28.5195, corrected p<0.001), DMN (T=4.3253 corrected p<0.001), Motor (T=21.783, corrected p<0.0001), SAN (T=-6.0826, corrected p<0.001), Subc. (T=-10.2842, corrected p<0.0001), VAN (T=-8.2092, corrected p<0.001) and Vis. (T=-13.2459, corrected p<0.001) differed from the average SD of functional connectivity of the whole brain.
For chunks of 180 seconds, ANOVA revealed a difference between networks (F(10,7073)=193.8473, p<0.001). Tukey's honest significance test revealed that FPN

(T=8.3367, corrected p<0.0001), Aud. (T=11.7284, corrected p=0.0024), CON (T=-9.3115, corrected p<0.0001), DAN (T=-25.5631, corrected p<0.001), DMN (T=2.662, corrected p<0.05), Motor (T=20.2498, corrected p<0.0001), SAN (T=-4.43, corrected p=0.0001), Subc. (T=-11.4505, corrected p<0.0001), VAN (T=-5.3993, corrected p<0.001) and Vis. (T=-11.8237, corrected p<0.001) differed from the average SD of functional connectivity of the whole brain. Results from chunks of 240 s are not shown as the movie clip used in CamCAN was too short.

Table 4
The average standard deviation across participants for each network for each chunklength calculated with a regression model is shown for: Frontal Parietal Network (FPN), Cingulo Opercular Network (CON), Salience Network (SAN), Dorsal Attention Network (DAN), Ventral Attention Network (VAN), Default Mode Network (DMN), Motor Network (Motor), Auditory Network (Aud.), Visual Network (Vis.), Subcortical Network (Subc.), Whole Brain (WB).

CamCAN								
	Reg GVC				Reg BVC			
	60 s	90 s	120 s	180 s	60 s	90 s	120 s	180 s
Aud.	0.2679	0.2137	0.1835	0.1354	0.2679	0.2129	0.1819	0.1339
CON	0.2387	0.1908	0.1631	0.1188	0.2399	0.1917	0.1645	0.1199
DAN	0.2148	0.1738	0.1480	0.1097	0.2122	0.1710	0.1450	0.1066
DMN	0.2512	0.2008	0.1712	0.1263	0.2515	0.2011	0.1713	0.1263
FPN	0.2537	0.2043	0.1737	0.1284	0.2557	0.2060	0.1752	0.1295
Motor	0.2680	0.2138	0.1824	0.1355	0.2724	0.2176	0.1859	0.1383
SAN	0.2409	0.1932	0.1650	0.1223	0.2425	0.1942	0.1662	0.1233

Subc.	0.2402	0.1888	0.1600	0.1166	0.2430	0.1917	0.1623	0.1187
VAN	0.2282	0.1848	0.1596	0.1209	0.2278	0.1841	0.1586	0.1195
Vis.	0.2417	0.1933	0.1632	0.1203	0.2420	0.1940	0.1638	0.1220
Whole Brain	0.2489	0.1991	0.1696	0.1253	0.2492	0.1995	0.1700	0.1256

2.4 Correlation results of Global-Network Variable Connectivity

For chunks of 30 seconds, ANOVA revealed a difference between networks (F(10,154)=127.9191, p<0.001). Tukey's honest significance test revealed that the FPN (T=-8.2087, corrected p<0.05), Aud. (T=18.9795, corrected p<0.01), CON (T=-3.9031, corrected p<0.001), DAN (T=-6.6094, corrected p<0.001), DMN (T=26.1438, p<0.001), Motor (T=-11.5163, p<0.001), SAN (T=-14.2855, p<0.001), Subc. (T=-7.0013, p<0.001) and Vis. (T=6.9391, p<0.001) differed from the average SD of functional connectivity of the whole brain.

For chunks of 60 seconds, ANOVA revealed a difference between networks (F(10,154)=110.6367, p<0.001). Tukey's honest significance test revealed that the FPN (T=-6.8493, corrected p<0.001), Aud. (T= 21.2923, corrected p<0.01), CON (T=-4.4409, corrected p<0.001), DMN (T=22.5487, p<0.001), Motor (T=-13.6927, p<0.001), SAN (T=-9.3708, p<0.001), Subcortical (Subc.; T=-10.7801, p<0.001) differed from the average SD of functional connectivity of the whole brain.

For chunks of 90 seconds, ANOVA revealed a difference between networks (F(10,154)=146.8832, p<0.001). Tukey's honest significance test revealed that the Aud. (T= 21.8089, corrected p<0.01), CON (T=-3.8206, corrected p<0.0001), DAN (T=8.4549, corrected p<0.001), DMN (T=19.6227, corrected p<0.001), Motor (T=-13.9098, corrected p<0,001) Sub. (T=-17.9999, corrected p<0.0001) and Vis. (T=-6.0033, corrected p<0.01) differed from the average SD of functional connectivity of the whole brain.

For chunks of 120 seconds, ANOVA revealed a difference between networks (F(10,154)=181.10488, p<0.001). Tukey's honest significance test revealed that the FPN (T=-5.4644 , corrected p<0.0001), the Aud. (T=23.7379, corrected p<0.01), CON (T=-5.6896, corrected p<0.001), DAN (T=11.5004, corrected p<0.001), DMN (T=17.0092, corrected p<0.001), Motor (T=-12.9931, corrected p<0.0001), SAN (T=-5.2984, corrected p<0.001), Sub. (T=-17.9249, corrected p<0.0001), VAN (T=5.8003, corrected p<0.001) and

Vis. (T=-8.8348, corrected $p<0.001$) differed from the average SD of functional connectivity of the whole brain.
For chunks of 180 seconds, ANOVA revealed a difference between networks ($F(10,154)=222.0556$, $p<0.001$). Tukey's honest significance test (Tukey, 1949) revealed that the FPN (T=-3.2036, corrected p= 0.0014), Aud. (T=19.9217, corrected p=0.0024), CON (T=-12.3139, corrected $p<0.0001$), DAN (T=15.0835, corrected $p<0.001$), DMN (T=14.323, corrected $p<0.001$), Motor (T=-9.9121, corrected $p<0.0001$), SAN (T=-5.1521, corrected p=0.009), Sub. (T= -20.6355, corrected $p<0.0001$), VAN (T=12.2013, corrected $p<0.001$) and Vis. (T= -9.5042, corrected $p<0.001$) differed from the average SD of functional connectivity of the whole brain. All results are available at https://github.com/chiaracc/FPNflexiblehubs/tree/main/res/camcan.

2.5 Correlation results of Between-Network Variable Connectivity

For chunks of 30 seconds, ANOVA revealed a difference between networks, $F(10,154)=98.6045548$, $p<0.001$. Tukey's honest significance test revealed that FPN (T=-20.9403, corrected $p<0.05$), Aud. (T= 22.3623, corrected $p<0.01$), DAN (T= 3.5612, $p<0.001$), DMN (T=26.1128, $p<0.001$), Motor (T=-8.0864, $p<0.001$), SAN (T=-4.9584, $p<0.001$) and Subc. (T= -4.3592, corrected p=<0.001) differed from the average SD of functional connectivity of the whole brain.
For chunks of 60 seconds, ANOVA revealed a difference between networks ($F(10,154)=81.843202830$, $p<0.001$). Tukey's honest significance test revealed that the Aud. (T= 22.3623, corrected p=0.0024), DAN (T=3.5612, corrected $p<0.01$), DMN (T=26.1128, $p<0.001$), Motor (T=-8.0864, $p<0.001$), SAN (T=-4.9584, $p<0.001$), Subc. (T=-4.3592, $p<0.001$) differed from the average SD of functional connectivity of the whole brain.
For chunks of 90 seconds, ANOVA revealed a difference between networks $F(10,154)=679.221136125$, $p<0.001$. Tukey's honest significance test revealed that the Aud. (T=22.6806, corrected p=0.0024), DAN (T=9.9544, corrected $p<0.001$), DMN (T= 22.4648, corrected $p<0.001$), Motor (T=-9.8857, corrected $p<0.001$), Sub. (T=-12.3307, corrected $p<0.0001$) and Vis. (T=-3.6656, corrected p=0.0045) differed from the average SD of functional connectivity of the whole brain.
For chunks of 120 seconds, ANOVA revealed a difference between networks ($F(10,154)=875.9554$, $p<0.001$). Tukey's honest significance test revealed that the Fronto-Parietal Network (FPN ; T=148.0011, corrected $p<0.0001$), the Aud. (T= 23.9698,

corrected $p<0.01$), DAN ($T=12.7994$, corrected $p<0.001$), DMN ($T=19.1233$, corrected $p<0.001$), Motor ($T=-9.3032$, corrected $p<0.0001$), Sub. ($T=-13.4548$, corrected $p<0.0001$), VAN ($T=6.7634$, corrected $p<0.001$) and Vis. ($T=-7.3281$, corrected $p<0.001$) differed from the average SD of functional connectivity of the whole brain.

For chunks of 180 seconds, ANOVA revealed a difference between networks ($F(10,154)=195.7896782$, $p<0.001$). Tukey's honest significance test revealed that the Aud. ($T=19.9323$, corrected $p<0.01$), CON ($T=-9.8661$, corrected $p<0.001$), DAN ($T=15.8272$, corrected $p<0.001$), DMN ($T=15.9433$, corrected $p<0.001$), Motor ($T=-7.3027$, corrected $p<0.0001$), SAN ($T=-3.3344$, corrected $p<0.01$), Sub. ($T= -17.3359$, corrected $p<0.0001$), VAN ($T=12.4739$, corrected $p<0.001$) and Vis. ($T=-8.3558$, corrected $p<0.001$) differed from the average SD of functional connectivity of the whole brain. All results are available at https://github.com/chiaracc/FPNflexiblehubs/tree/main/res/camcan.
